# Supplementary material for: PhyloNaP: a user-friendly database of phylogeny for natural product–producing enzymes
Source: Bioinformatics. 2026 Jun 16;42(7):btag393. doi: 10.1093/bioinformatics/btag393 (PMC13344837; doi:10.1093/bioinformatics/btag393)
Supplement: btag393_Supplementary_Data [file btag393_supplementary_data.pdf]

Table of Contents

Dataset generation workflow *1*
Data sources 1
Datasets generation........ 2
The rooting method........ 3
Tree placement validation........ *4*
Database functional overview *22*
Functional categories from eggNOG........ 22
Superfamily-based overview. 22
MiBiG database coverage 23
A workflow example........ *24*
References *29*

Dataset generation workflow

The dataset generation pipeline consists of a series of automated steps designed to collect, filter, and organize protein sequences into phylogenetically structured datasets.

Data sources

Protein sequences were collected from several established resources. The MiBiG 4.0 database (Mitja M Zdouc et al. 2024) provided proteins from experimentally characterized biosynthetic gene clusters (BGCs), while MITE (Mitja M. Zdouc et al. 2024) contributed enzymes with experimentally validated reactions. AntiSMASH-DB 4.0 (Blin et al. 2024) supplied predicted BGC-derived proteins from large-scale genome mining, and UniProt SwissProt (Boutet et al. 2007; The UniProt Consortium 2025) added manually curated sequences with high-quality functional data.

Each sequence was enriched with metadata. Taxonomic information was retrieved via the NCBI Datasets Taxonomy Data Package (O'Leary et al. 2024). Functional annotations, BGC product classes, and cross-links to external databases were added where available. Structural data for substrates or products were obtained either directly from MITE and MiBiG or indirectly via UniProt cross-references to ChEBI (Hastings et al. 2016) and Rhea (Bansal et al. 2022). RDKit (Landrum et al. 2020) was used to generate chemical structure depictions. Sequences from antiSMASH-DB were additionally mapped to PanBGC (Paccagnella et al. 2025) to enable cross-referencing. Set of dereplicated sequences (<70% identity) was also annotated with EggNoG and domains from the Superfamilies database.

Datasets generation

Sequences were clustered into datasets using MMseqs2 (*easy-clust*) (Steinegger and Söding 2017) with minimal length 80 and sensitivity 7.5 (the most sensitive mode), generating quite broad clusters, with relatively high diversity. Several filtering steps were applied to ensure biological relevance and computational feasibility. Clusters containing only SwissProt entries (missing biosynthetic context, irrelevant), clusters with extreme mean sequence lengths (<70 amino acids).

For each retained cluster, multiple sequence alignment was performed with MAFFT (auto mode) (Katoh and Standley 2013), followed by automatic trimming with TrimAl (Capella-Gutiérrez et al. 2009). Identical sequences were removed after trimming, while unique annotation information was retained in dedicated metadata fields in columns <original column name>_others. Clusters reduced to fewer than 10 sequences after this step were discarded. Phylogenetic trees were inferred using FastTree (Price et al. 2010). Because sequences with low pairwise identity (<30%) may have been grouped within the same cluster during the initial clustering step, additional iterative filtering was performed to improve tree quality. Evolutionary diversity within each tree was assessed using TreeCluster (Balaban et al., 2019), which partitions phylogenetic trees based on evolutionary distances between clades. Based on empirical evaluation, a threshold of 4 was selected as the maximum allowable pairwise distance between leaves within each cluster. Trees exceeding this threshold were subdivided into subclusters; each subcluster was then realigned and trimmed. Following this step, alignment length retention was calculated as:

After this step, alignment length retention was calculated as:

alignment length retention= $\frac{length\ of\ the\ trimmed\ alignment}{mean\ sequence\ length}$

Alignments with values <0.75 were discarded due to insufficient post-trimming length. Alignment quality was further evaluated using AliStat. Alignments were excluded if row completeness (Cr; median proportion of fully specified characters—excluding gaps and ambiguous residues—per sequence) was <0.7, or if overall completeness (Ca; proportion of fully specified characters across all alignment positions and sequences) was <0.7. These steps ensured high-quality alignments.

While curated datasets rely on evolutionary models specifically selected for each case, large-scale automated inference requires a faster and more uniform approach. FastTree, which is well suited for generating thousands of trees efficiently, applies the JTT (Jones–Taylor–Thornton) model of amino acid substitution by default. Accordingly, all automatically generated datasets were inferred under the JTT model, ensuring consistency and comparability across the collection.

The rooting method

Phylogenetic trees were rooted using a custom taxonomy-based algorithm that identifies biologically meaningful root positions based on outgroup detection. For each dataset, the method takes an unrooted tree together with taxonomic annotations and evaluates all possible bipartitions to identify candidate roots.

The algorithm operates hierarchically across taxonomic ranks (superkingdom to species), prioritizing higher-level classifications. At each level, the dominant taxon (i.e., the most frequent annotation) is determined, and candidate root positions are evaluated using two complementary criteria: (i) detection of a pure outgroup, defined as a clade composed exclusively of a minority taxon distinct from the dominant group, and (ii) dominant monophyly, where rooting at a given position results in the dominant taxon forming a monophyletic group while the opposing clade lacks members of this taxon. To ensure robustness, several constraints are applied. Candidate outgroups must contain at least two sequences and exhibit sufficient taxonomic annotation coverage (≥70%). Additional safeguards restrict rooting at lower taxonomic levels (order, genus, species) to cases with adequate global annotation coverage and clear taxonomic dominance, preventing spurious inference from sparse or ambiguous annotations. Furthermore, rooting at shallow taxonomic levels requires stronger phylogenetic support, implemented as a minimum branch length threshold relative to the tree-wide distribution. When multiple valid root positions are identified, candidates are ranked by (i) taxonomic level (favoring higher levels), (ii) outgroup size, (iii) outgroup purity, and (iv) branch length support. The highest-ranking candidate is selected as the root. If no candidate satisfies all criteria, the tree remains unrooted.This approach was applied to all automatically generated datasets and successfully identified taxonomically consistent roots for approximately 65% of trees. For the remaining datasets, rooting was performed using the minimal ancestor deviation (MADroot) method (Bryant and Charleston 2018), ensuring complete coverage.

Each dataset was assigned to an enzyme superfamily based on HMMER searches against the Superfamilies v1.75 (Pandurangan et al. 2019) profile set. All detected domains were aggregated to generate a dataset-level superfamily annotation. Datasets with identical superfamily combinations were grouped together. All datasets and associated metadata were stored both as a structured JSON index and in a NoSQL database, enabling efficient access and filtering. For each dataset, summary statistics were compiled, including the total number of sequences, the number of experimentally characterized enzymes (from SwissProt with Rhea annotations or from MITE), the number of proteins derived from validated BGCs (MiBiG), and the number of proteins from predicted BGCs (antiSMASH-DB).

This procedure results in a collection of high-quality, phylogenetically structured datasets optimized for functional exploration and user-guided sequence placement.

Tree placement validation

Reference tree from literature	Query sequence used	Result
Hansen et al. 2023 Supplementary Information Figure S19	>AmAl_PCGEMLEO_00020 MGDQAVRTAHSDVLLASAGERGVLCDFYGEAAAD TYRDLVEDGDGTSEAQAFAARVHPVSAPVLELAAG VGRLSFPFLEFGWELTALELSATVVAAFRERLAKEP ADVRDRCTVVQADMSAFSLGKTFGTVVISSGSINEL DEYDRSRVYAAVREHLDPGGKFLLSLELGKPGEPEP MERRQELTGRSGRRHALHVKVVPSEEIQEITIYPADE TADPFVVCTHRRRLVPSDRTVRELVRAGFDVISQTPF ASSEAGREDMLLVEAVTPAVR	Correct placement with methyltransferases from other GPA clusters.<br><br>From the product descriptor it is possible to deduce its function as N-methyltransferase.<br><br>Figure 1
Hansen et al. 2023 Supplementary Information Figure S19	>AmSB_2_extraction_OPILMPDP_00024 MQESLDSTEVHRRFQLIANGPALFNAVVSGIELDIFD FLAEKDGADAAELGAFTGLEPHKLRVLMLALATTG LIEKRGVDFVNHPVATELLAASGPESWRHILLSWKTI YYPAFARMTSALRAGTNEALDALDGPGDTLYARLG NDPEKARIFYTAMSAFSLQTMPGLLEHIDVKSSRHLL DVGGGDGTTAAALLKANPGLKATLLDLPSSVALAE QRVPAEIVDRLTLHPADLLTDPFPTDTDHALFSHVLD TFTAEQSVMLLAKAYEVLPSGAKISIYGFAAADDET GGPLSARLSLYLNILATGRGMAWPQAEAASWLKSV GCVDVRSVELPFEHALVTGTKP	Correct placement with methyltransferases from other GPA clusters.<br><br>The type of methylation remains unknown, and no specific substrate can be inferred from their phylogenetic placement.<br><br>Figure 2
Hansen et al. 2023 Supplementary Information Figure S19	>SsW1420_2_extraction_FCIEGDNP_00015 MPENGLFTSTVALSAQPDRMCVKAGAVNGEEGFPL SLDMLLILDIMLHEHDLAAAEAQFTAVRKAPLAADA QAAVDELSYSGNTCSIHARRGTLTVRDDRKMVRAA DLTAQSRSTRQVLRALGMRRNLSHDPETPNFRELFT ELQERGLLVPSIGEIEWGQLRRLTPICPDFGISRGTPID RHYLNQFIAEIRDLVQGDVVEIGGNDGNKDVYGFTR TSGYRGLDINDAPGVSLVGDAADPGIIPADSLDTIIAF NVLEHTARPWQVVDNMRQWLRKGGTAYCMVPSA	No tree building possible using this query.<br><br>No reference tree was found.<br>Fewer than 10 homologous proteins in the PhyloNaP dataset.

	QRLHGAPEDYWRPLPAALREMFGAWSEQTVYQYG<br>NPLSVIASLMGIAAEELDALELSTYHPDYPVASCIVA<br>RK	
Hansen et al. 2023<br>Supplementary Information<br>Figure S19	>AmL_extraction_LBFIIEIF_00021<br>MSDLAAAPHENRQMAQWFGADAERYDRARPTYPE<br>ALVDRVVSLSPGKNFVDVGCGTGLSSRPFQAAGCTV<br>LGVEPDARMAEVARRRGLDVEVAKFEDWDPAGRT<br>FDAVTSGTAWHWVDPFAGAAKVADVLAPHGLIALF<br>DNGFELPEAVMTAQGEAYRHAMPDVKFEPPSKKDE<br>DVEEYVQQVYAKQYVKAAEGILRTGAFSRPEELRFE<br>WSRVCTRDEWLDQIPTQGGLNHLAEDARARFLAYL<br>GSAIDEIGGSFTINYATVGIAAVRR	Correct placement with methyltransferases from other GPA clusters.<br><br>The type of methylation remains unknown, and no specific substrate can be inferred from their phylogenetic placement.<br><br>Figure 3
Hansen et al. 2023<br>Supplementary Information<br>Figure S19	>AmAl_PCGEMLEO_00018<br>MSTTSRCRICDGTVTEFIDLGRQPLSDAFVVPGDEKK<br>EFFFRLATGICESCTMVQLMEEVPRDLMFHEAYPYH<br>SSGSAFMRTHFHDLAKRLLTTELTGEDPFIVELGCND<br>GIMLKAIAEAGVRQLGVEPSGGVADLAAAKGIRVR<br>KDFFEEATAADIRENDGPADVIYAANTLCHIPYMDSI<br>LKGVTTLLGPNGVFVFEDPYLGDIVERTSFDQIYDEH<br>FFFFTARSVQEMAKRNGLELVDVERIPVHGGEVRYT<br>LALAGARKPSEAVAELLAWEAERKLSEVATLERFA<br>ANVKKIKEDLIALLTKLRAEGKRVVGYGATAKSAT<br>VTNFCGITPDLVEFISDTTPAKQGRLSPGQHIPVREYR<br>EFAGDHPDYALLFAWNHADEIMNAEQGFRDAGGQ<br>WIRYVPNVHVS	Correct placement with methyltransferases from other GPA clusters<br><br>From the product descriptor it is possible to deduce the enzyme function as C-methyltransferase.<br><br>Figure 4
Hansen et al. 2023<br>Supplementary Information<br>Figure S13	>AmAz2_extraction_LNHOCGCG_00025<br>MRVLLSTSGSRGDVEPLVALAVRLRELGAEVRMCA<br>PPDAGERLAEFGVPLVPVGESTRAMTHEKKPPSPKD<br>GPRLSAEAIATQFEQVPAGAEGCDAVVATGMLAAA<br>VAVRSVAEKLGIPYFYAFHCPIYLPSPHYPPPPPLGEP<br>PAPAGTDIRSLWARNSQSAYRRFGEPLNSERAAIGLP	Correct placement of the glycosyltransferases among other GPA glycosyltransferases homologous to GtfB.<br><br>Figure 5

	PVDDIFSYGYTDHPLLASDPVVAPLLPTDLDAVQTG<br>AWTLPDERPLSPELEAFLAAGEPPVYVGFGSMRAPE<br>DAAKVAVEAVRAQGRRMILSRGWADLTLIDDQADC<br>FAVGEVNHQVLFGRVAAVVHHGGVGTTTAAARAG<br>APQVVVPQIADQPYFAGRVAELGIGAAHDGPTPTVD<br>SLSAALTVALSPETRARAAAVAGTIHHDGAAVAAK<br>LVLEAVNRQGSTERGTL	
Hansen et al. 2023 Supplementary Information Figure S13	>AvH5_extraction_OMOLLKKM_00019<br>MVWRSPAGQPGWARPALLAIAAVAAVLHAWNLPR<br>ADFAPLYSQAVKGMSESWKALFYGAIDANATVTLD<br>KLAGSFVPQAVSARIFGYHAWSLALPQVIEGVISVLV<br>MYRVVRRWAGVVPGLLAAGIFTFTPVAASMFGHSM<br>EDGALVMCLVLAADAYQRAVMEARLRSLVWAGV<br>WVGLGFQAKMLQAWMILPALAIGYLLAAPAGLRRR<br>LGHLAVAGVVTIAVSLSWIALYTFTPAGARPAINGTT<br>NNSAFTMVFGHNGLSRLGIALPGTVPNNGRVRAVV<br>GPGQHLTPGELNGEGLPGAPQGPAGTPGNGPAAAPG<br>AGPEADGGAAKLLAGRLGIAIGWLYPLTLLALLCGL<br>WWRRHAERTDPERGGLVMWGVWLLTFGLTFSATD<br>VPHTAYVASLAPPVAALAGLGIVRFSQAYRRGGRA<br>AWLLPAAVLAELVWAAWLWSDYLGFLPLALLSTLA<br>LGVAAIVVLVLGRVGKPASAAVVRAGLALGAAAIL<br>AAPVTYAASVLGPAYSGNSFDANAGPVKVEGVPQG<br>G	Correct placement of the glycosyltransferases among other GPA glycosyltransferases homologous to GtfD.<br><br>From the product descriptor it is possible to deduce its function as mannosyltransferase.<br><br>Figure 6
Hansen et al. 2023 Supplementary Information Figure S13	>AmSM_extraction_CJOCMKKM_00035<br>MTTDGLQPSVDPSNDNAFLRWVRKHQRWLYAGVV<br>IALLTQMAIAMITTAVEQSPTIDEPVYVGTAVVYLEE<br>HSLRYNPEHPPLGKLIIAAGVAFTDPKIAPGFSGHEW<br>ELGKHVVYEAGNDPDRLMLAARLPVILLTLLFGLVV<br>FAFASDLAGRAGGVLALTLFTFSPDVIAHGSLATLD<br>VPVAGFLLTSVWLVWRARRRPGLYLPLAGLALGAA<br>TATKVLSLVVVPVLIPLAVVSVWHARRARGLDLKSP<br>ALLIGRGVLAGAGMALIAIVVVWASYLVVDPGLRW	Correct placement of the glycosyltransferases among other GPA glycosyltransferases.<br><br>From the product descriptor it is possible to deduce its function as mannosyltransferase, but it is placed in a different tree than the mannosyltransferases in Figure 6.<br>Recent literature shows that, while required for mannosyl incorporation into the GPA, this

	ETPSGMQPLHGMRTLVEWLPFPQPLRDGMRFQFELE<br>DHVWHNFLFGRHYTGSLWYYGPAALLVKTPLGAL<br>AIWLAGAAVLLSVRRLRPAAPYVLVPTALLMATAM<br>VIVRDNGVRYLAFAPMFLAVAAAAVLVVRRRWVK<br>VAAVAVVAFAAVSSLMTFPYYLPYSNEAFGGPENT<br>HKYLLDSNVDWGQDLGRLADRLKQDYAGERTWLV<br>YQGSGLPSYYGIDAADPLTVPEDQVHGVLAVSDSAV<br>LGAFADPMGGGGAPGNGHGSDLGSLIAGSTPVERV<br>GHSMTIYRRP	enzyme has a more diverse function than that of a simple mannosyltransferase (Cui et al. 2025). This observation fits with the placment in a different tree.<br><br>Figure 7
Thomy et al. 2019<br>Supplementary Information<br>Figure S8	>AdeA_L-leucine-5-hydroxylase_ADEP<br>VQLAKSVLDQYDRDGFVLVPGAFSPAEMDCLKGA<br>MAEDVASSKGPHLITEDDGATLRAVYASHTRHPLFS<br>TLVSSARLLA<br>PAMQLVAQDLYVHQFKINTKRPFGGESWAWHQDY<br>PVWRDADRMPEPRAVNVAVFLDEVTEFNGPVVFLR<br>GSHRLGSEASS<br>RQQANQAGEHIDPHDYALSTGDLSKLAEVHEMTSP<br>KGPAGTVVFFHPEIMHGSAPNISPFPRDLLIVTYNAST<br>NAPRPVG<br>EPRPEYLVGRDVTPLVPNSWTLETIGASIARAES	Correct placement of AdeA with the leucine hydroxylases.<br><br>Close relationship to the functionally related GriE from *Streptomyces muensis* could be confirmed.<br><br>Figure 8
Thomy et al. 2019<br>Supplementary Information<br>Figure S10	>AdeB_leucine_dehydrogenase_ADEP<br>MTTWEFAAAAVLEGFGQTVSIRKLRVPEPNEGEML<br>VDVTYGGICGTDLHLQQGHLPIPTPLTLGHEGLGTV<br>RSLGAGTTVDACGATLHVGDTVMWASSISCGQCMP<br>CRQYREPTLCEARRTYGVNRSLAEGAGLSGAWAETI<br>LLHPGVVVVKLPQGADELAAMSLACAGPTLVHALY<br>ERRPVRLGETVIVQGSGPVGLAAAALAHMAGAEKV<br>ILVGGPRQRLDLAAKCGIGHHHIDIVDGANPQQALD<br>QARALTPGGTGADLVIECAGIPGAVAQGLTLARRGG<br>SYLVVGQYTDNGDTMLNPHQIVHRQLDIHGSWAFS<br>GAHLVEYVRLLPVLSNRFDLRSLVVPFPLADVRIAM<br>EAVANGTVIKAVLQSA	Correct placement of AdeB with the leucine hydroxylases.<br>Close relationship to the functionally related GriF from *Streptomyces muensis* could be confirmed.<br><br>Figure 9
Grocholski et al. 2019<br>Figure 2	>EamK_WP_245240010.1	Correct placement with other enzymes from the reference tree.

	MSGTRNGTGGGADREQRPADDGPVEERPVDALVR MSNLVTPMALRVAATLRLVDHLRRGVTSPEALARA TGADADALSRLMRHLAAAGVLEETAPGRYAPTRLG DLLAEDDPSRQRSWLDLDQAVGRADLTFLDLREAV RTGRPQYEDRYGKPFWTDLSEDAELGASFDTLMTT REDAAFAAPVCAYDWSRARHVLDVGGAPGGLLSAI VRAAPGARGTLLDLPGAAQRTRERIAAAGLDDRIDV VAGDFFDELPVSADAVVLSFVLLNWSDADALRILAR CRDALRPGGRIVLLERAEAPSGGTRTSDLYFSVLDM RMLVFLGGRVRTDREWAALADAAGLRIVSRSGPLV SPSVPLDSCLWELAPH	Functionally distinct clades described previously were also resolved in the PhyloNaP dataset.<br><br>Figure 10
Harper et al. 2024<br>Figure 3	>FtdC_ EFL02195.1<br>MAGVPAARPGAAAQAPATDWESREPTPGTPPRVLVI GAGLAGLAAGSYGRMSGLRTLVLEKHVLPGGCCTA WSREGYVFDYCIEWLIGTAEGNEAHQVWRELGALD GKSVTNFELFNKVVGRDGREVVFWNDPDRLEAHLL ELSPADAPHIRAYCRDLRRFQKIELYPFLTAPALKTL GEKLRTLRTVLPAFRLFWRNAATPMHAFADKFQDP LLRTAFRNIFFQDPEGFPVLPYLFNMASAYHGNAGFP QGGSLGLARSVEERYTGLGGEIRYRARVERVLVEDD RAIGVVLRGGKTLYAEHVISAADGDTTIKGLLGGRY TGPRIDKLYEELLDQEGTLFPAVVSAFVGIEGGLPEG DAHSTTYLLDEQDAARLPGGLQSSIVVQLRSRYADG FAPEGKSVVHCTYFSDYGYWADLRAKDRRAYRAR KSEVAAFVREFLERKWPEVAGRIELVDVASPATTRR YTGNHKGSILAWKAFSEADDISAKLVGKDRMRLPG LAGFSMAGQWVGMGGLIRAASTGRFAVQYLCDEL GLPFRAFESENTEPWHPGKLGSLPQLDRWNEREERG R	Correct placement with other PhyDH enzymes in the PprB clade.<br><br>Correct phylogenetic distinction of PprA[5] (with PprA[5/6] subclade containing IkaB) and PprB. Polycycle prediction possible.<br><br>Figure 11
Harper et al. 2024<br>Figure 3	>FtdD_ EFL02196.1<br>MLIIGAGLGGLSTGVYAQLNGYRSRVLEMHEIPGGC CTAWERGDYTLDACVSWLLGSGPGNEMHQIWLEL GALQGKEVRHFDVFNVVRDTDGRAVYFYSDPDRLQ AHLTGISPADARHIKGFCDNLRSFRKALAVYPFLKPV	Correct placement with other PhyDH enzymes in the PprA[5] clade.

	GLMGRVERMRMLAGFLPYFNAVRKTISVLMRDYSE<br>KFQSPLLRRAFNYVLYERHPNFPVLPTHFQMASHAN<br>LSAGVPEGGSLGLARSIEERYVRLGGEVSYNTKVEE<br>VLIEGDTAVGVRLSDGTELRADIVVAACDGPTTMRS<br>LLKGRDLGKEYERLYTDTIEKPGMVFPGYVTLFLGL<br>KREFPEADPCTTYLLDDATAERLTGIRHSSINVQFRN<br>RHYPELSPPGTSVVYATYFCDIAPWRALDDGPEQRT<br>RRRGGEELHTLPVKHGRGYYAAKHQVRDTLVEFLE<br>ERFPGLSESIAVRDVSTPLTQVRYTGNFDGTVLGWQ<br>PFVESGETLEELIKKYGPGVPGLGNFYQSGVWATTG<br>GLIRAAAAGRHVMQFVCRDDGKEFTASVDTHGPLP<br>THRVIPVGPRVPGPAAQAAGGGVASLPVPPKLVPQK<br>EESR	Correct phylogenetic distinction of PprA^{5} (with PprA$^{5/6}$ subclade containing IkaB) and PprB. Polycycle prediction possible.<br><br>Figure 12

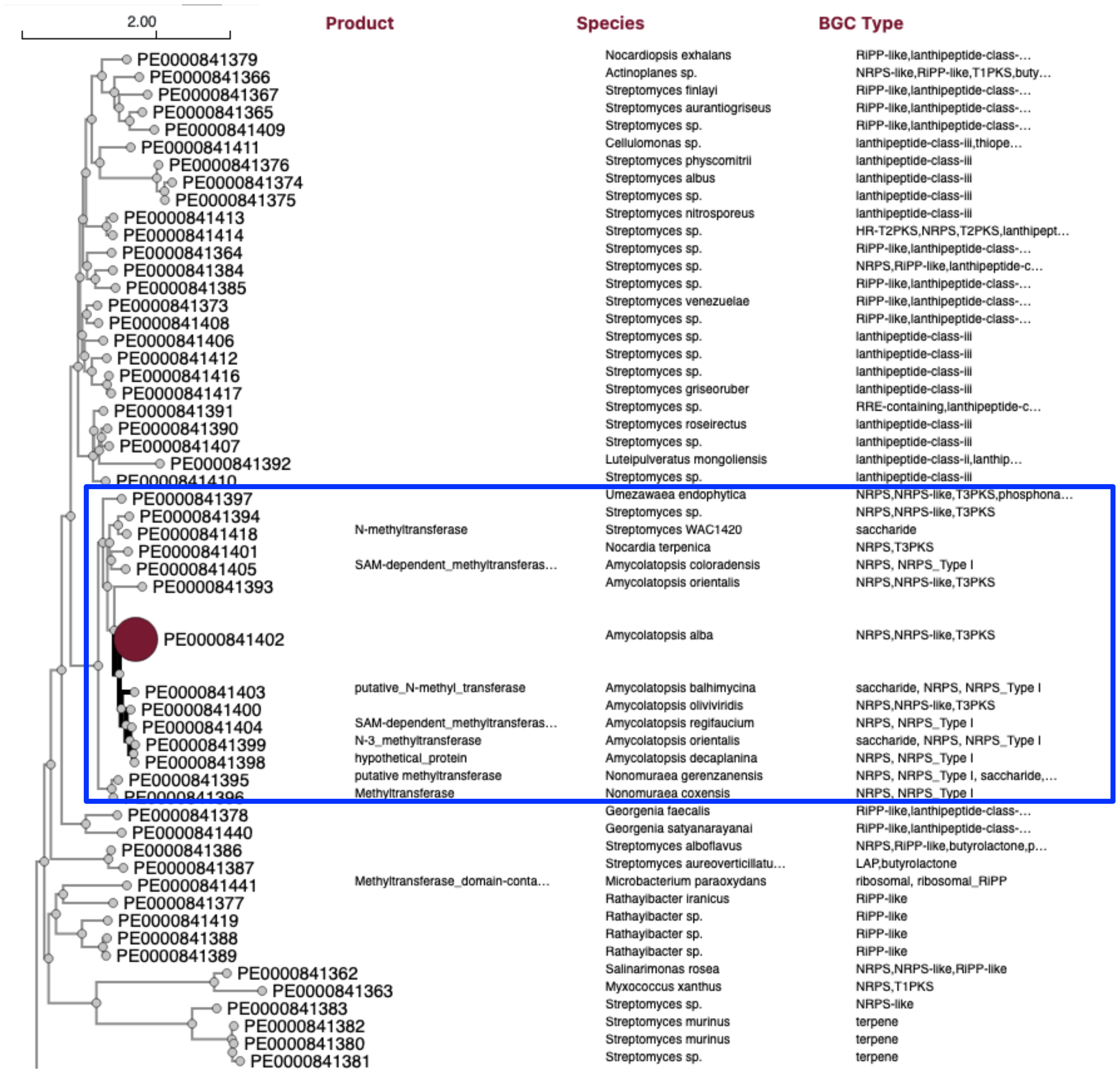

Figure 1: Detail from tree placement of GPA N-methyltransferase AmAl_PCGEMLEO_00020 in PhyloNaP. GPA methyltransferases highlighted in blue box.

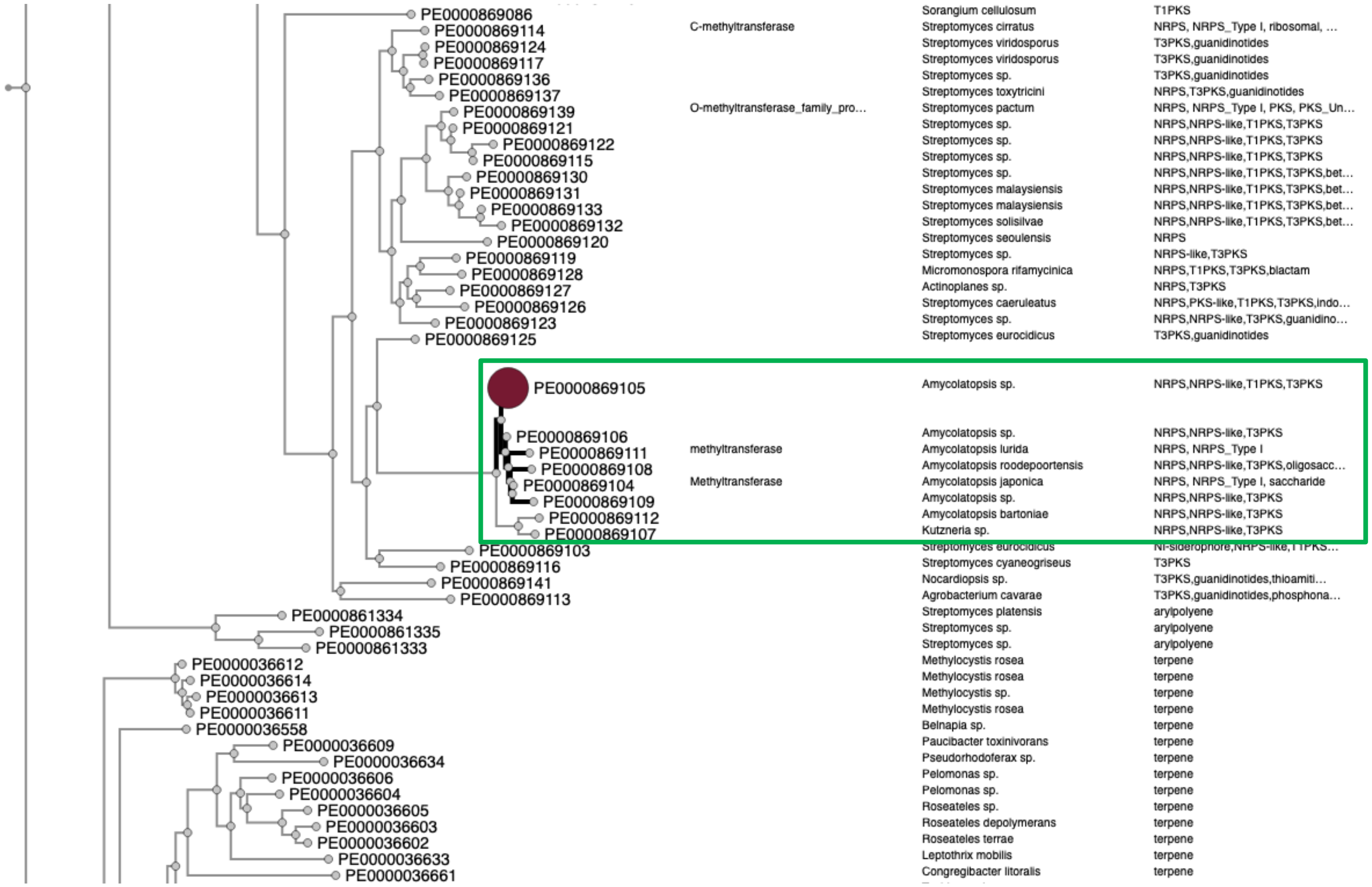

Figure 2: Detail from tree placement of GPA N-methyltransferase AmSB_2_extraction_OPILMPDP_00024 in PhyloNaP. GPA methyltransferases highlighted in green.

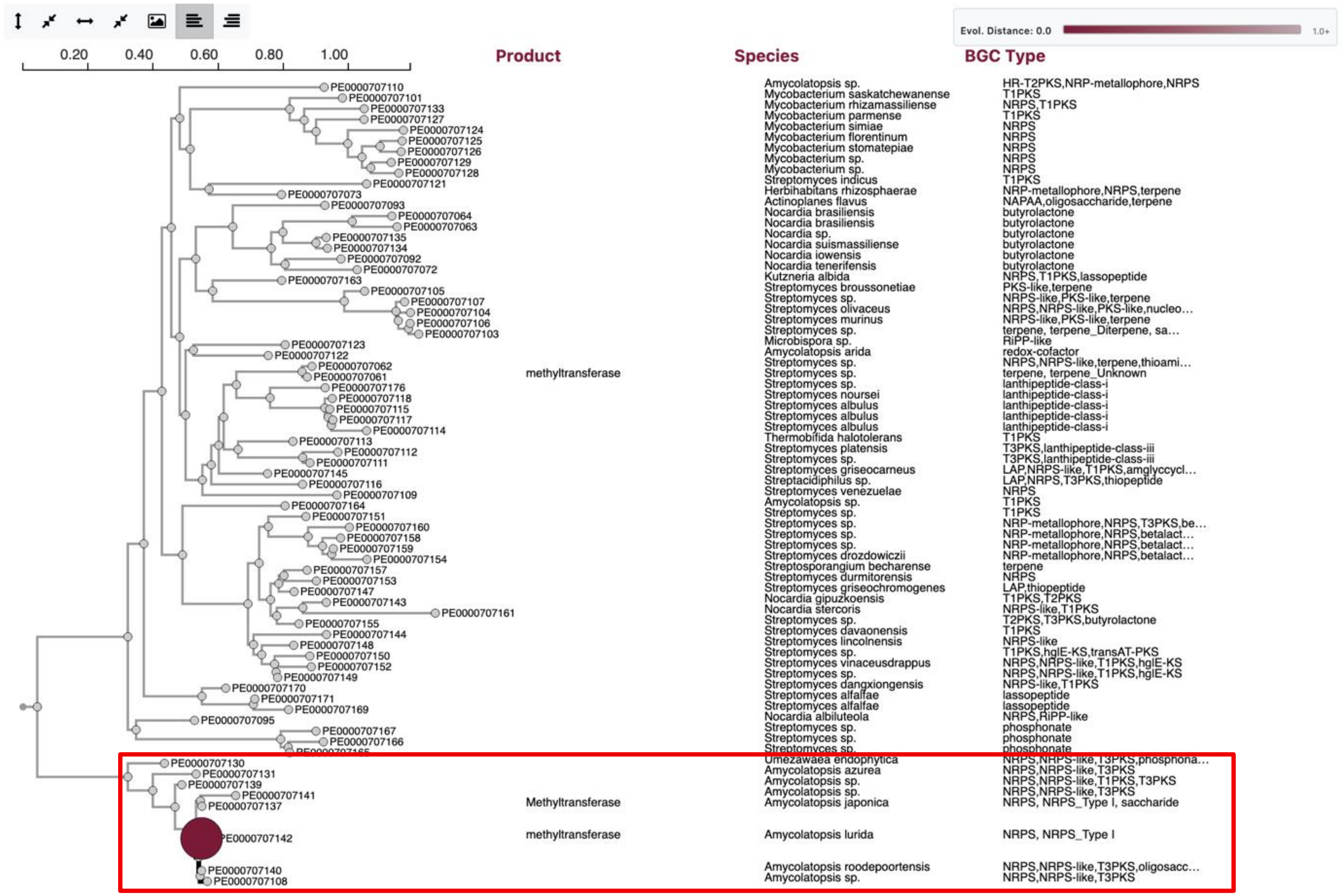

Figure 3: Detail from tree placement of GPA N-methyltransferase AmL_extraction_LBFIIEIF_00021 in PhyloNaP. GPA methyltransferases highlighted in red.

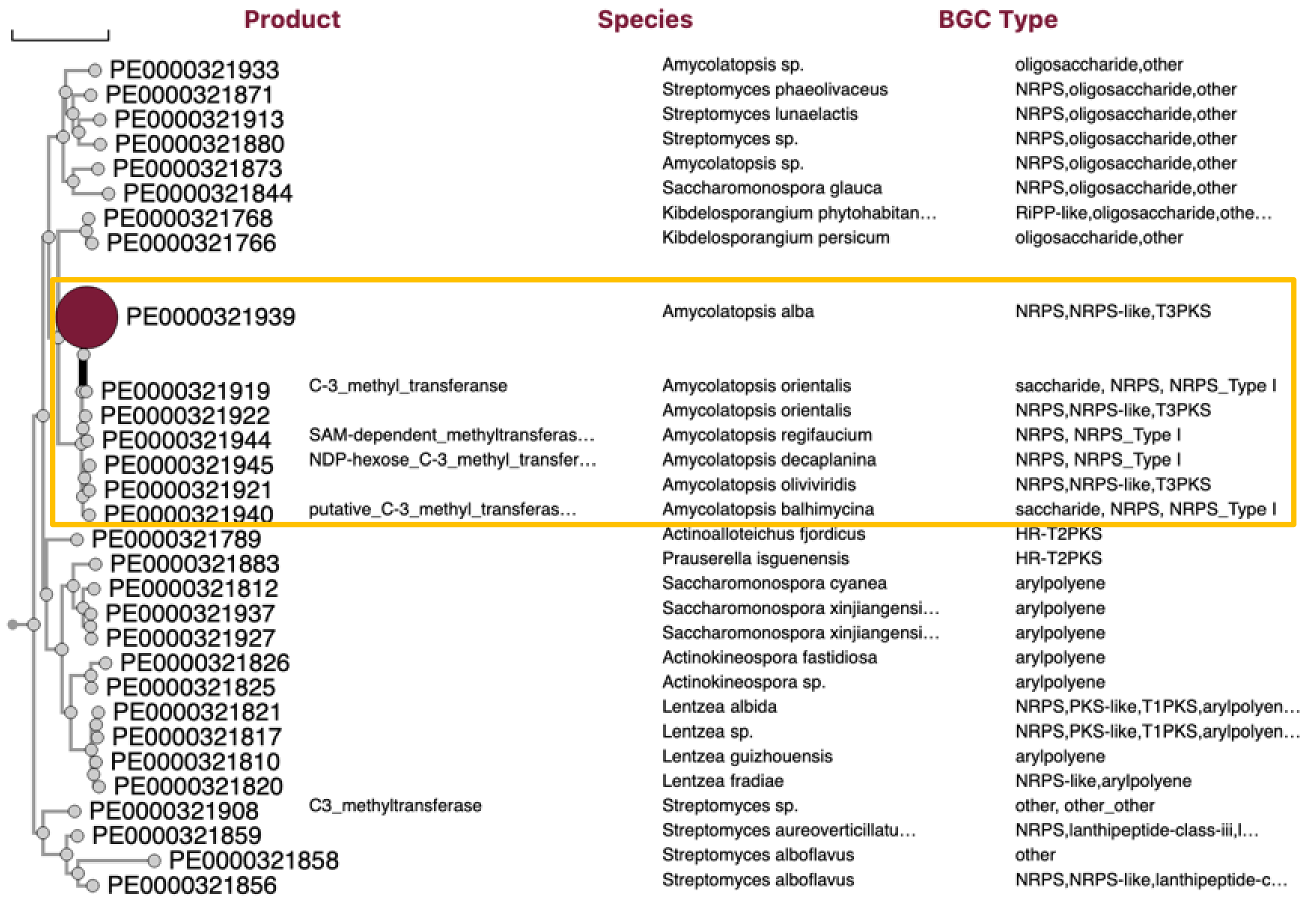

Figure 4: Tree placement of GPA C-methyltransferase AmAl_PCGEMLEO_00018 in PhyloNaP. GPA methyltranferases hightlighted in orange.

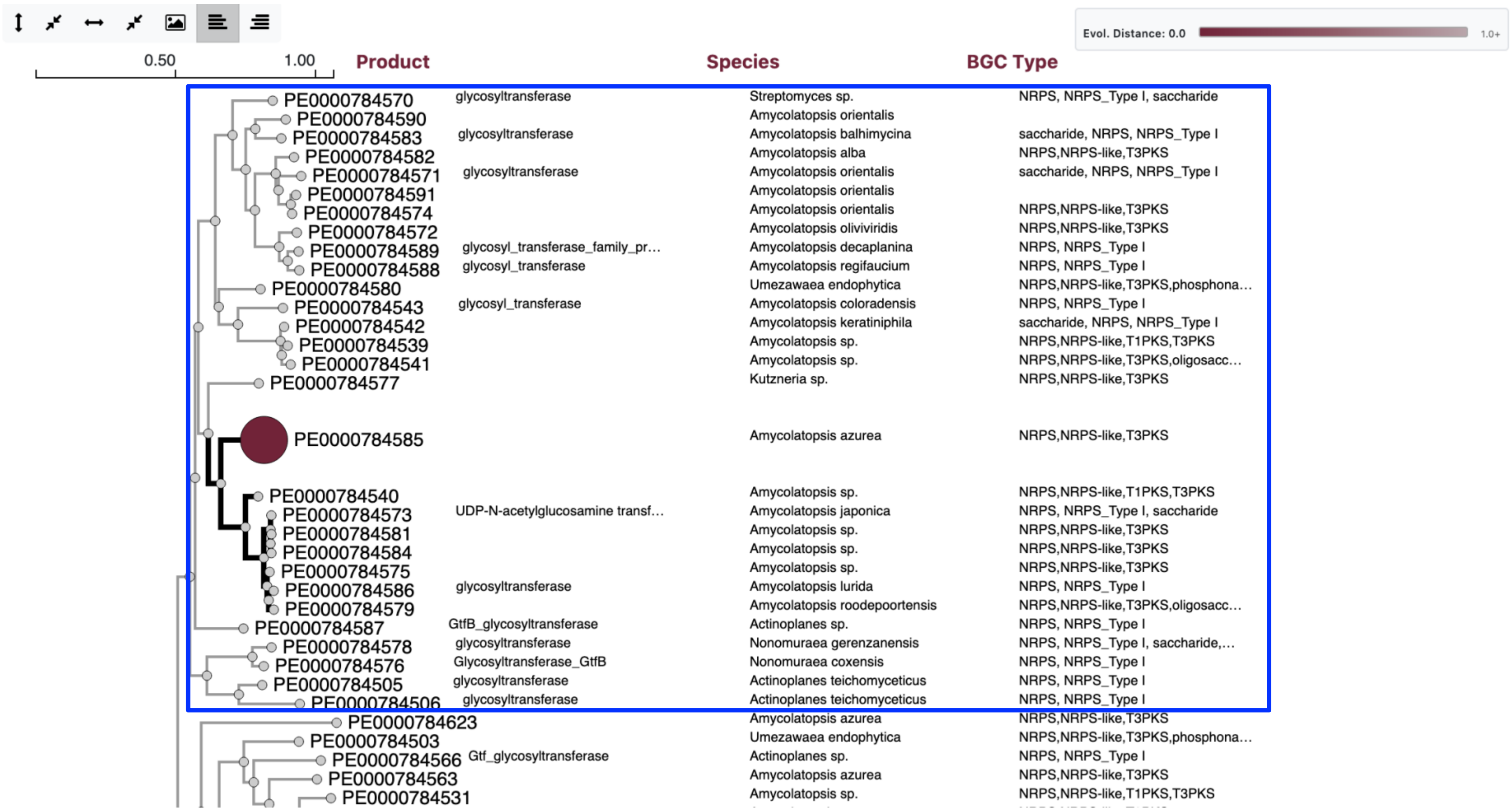

Figure 5: Detail from tree placement of GPA Glycosyltransferase AmAz2_extraction_LNHOCGCG_00025 in PhyloNaP. GPA glycosyltransferases (clade 8 from lit) is highlighted in blue.

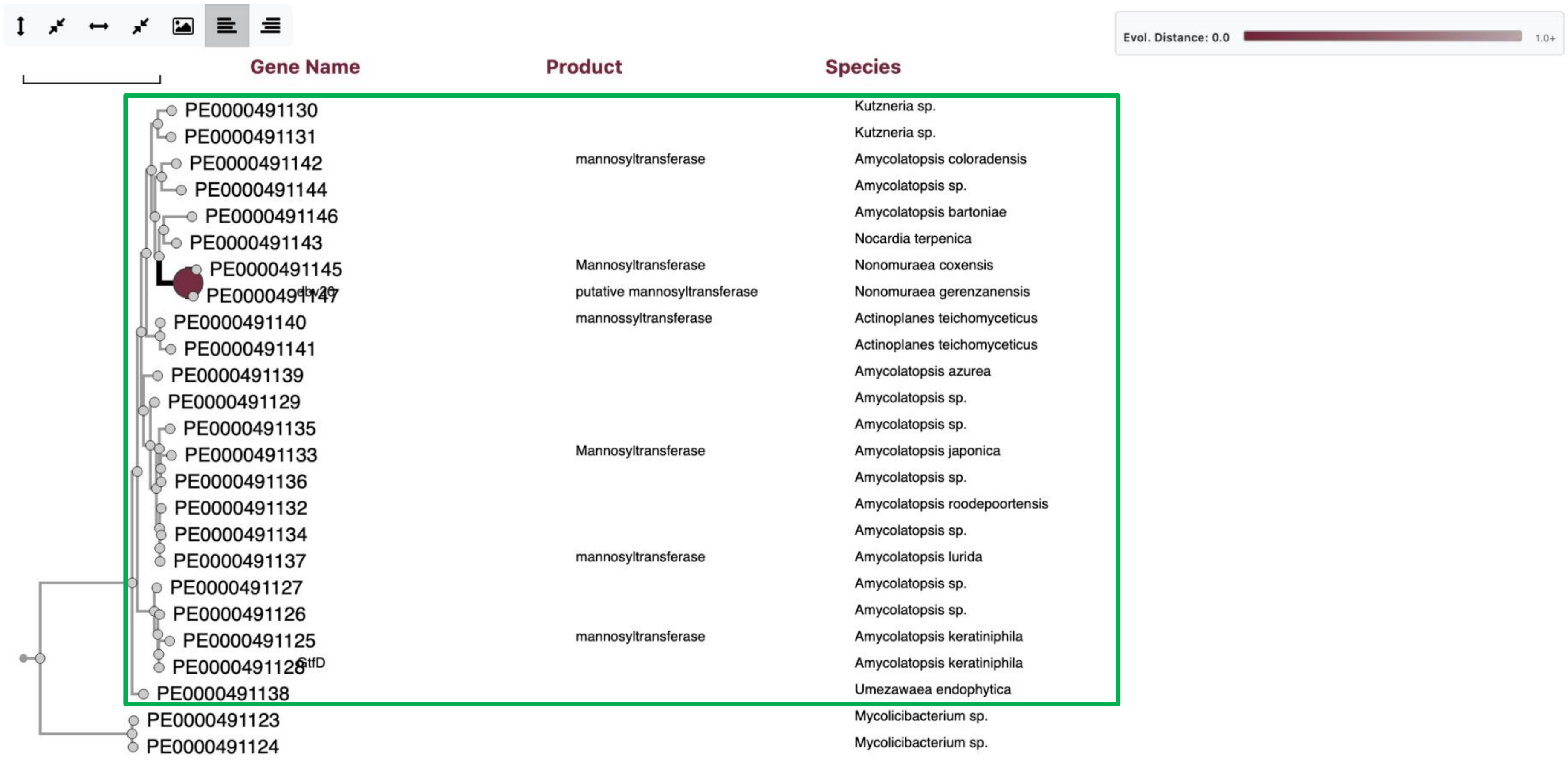

Figure 6: Tree placement of GPA Glycosyltransferase AvH5_extraction_OMOLLKKM_00019 in PhyloNaP. GPA mannosyltransferase clade is highlighted in green.

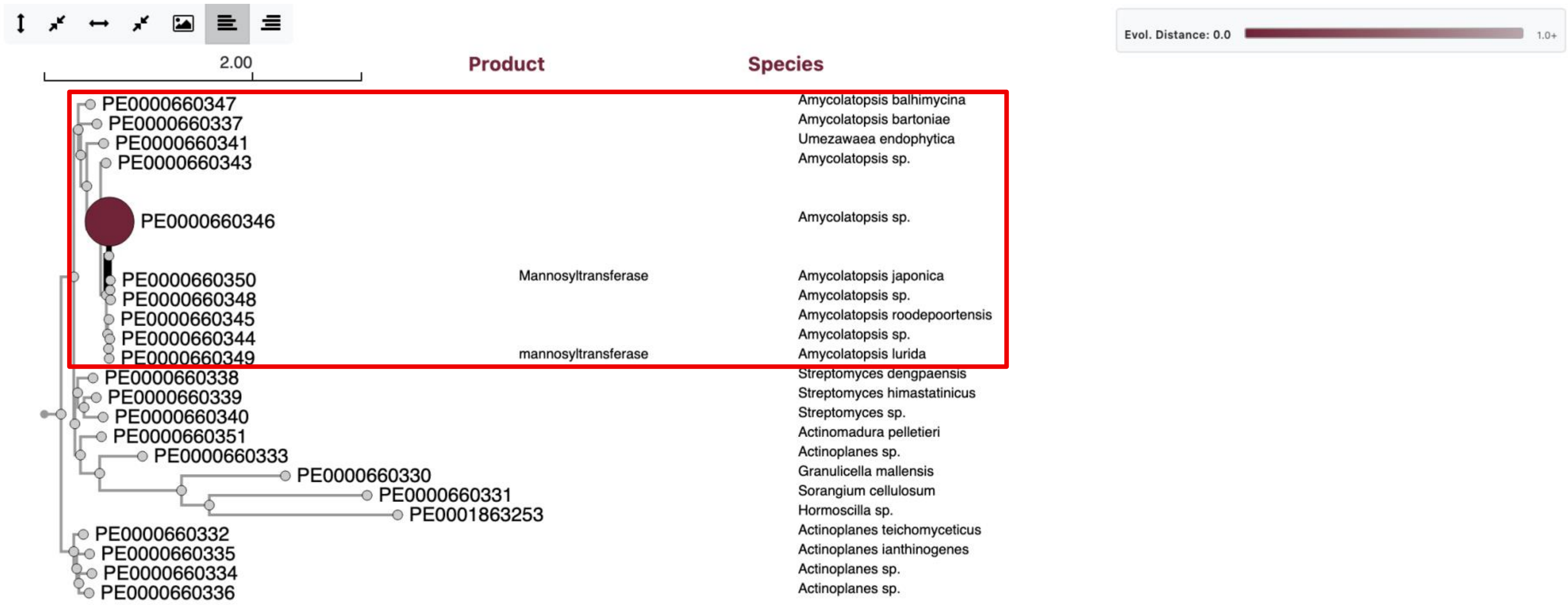

Figure 7: Tree placement of GPA Glycosyltransferase AmSM_extraction_CJOCMKKM_00035 in PhyloNaP. GPA mannosyltransferase clade is highlighted in red.

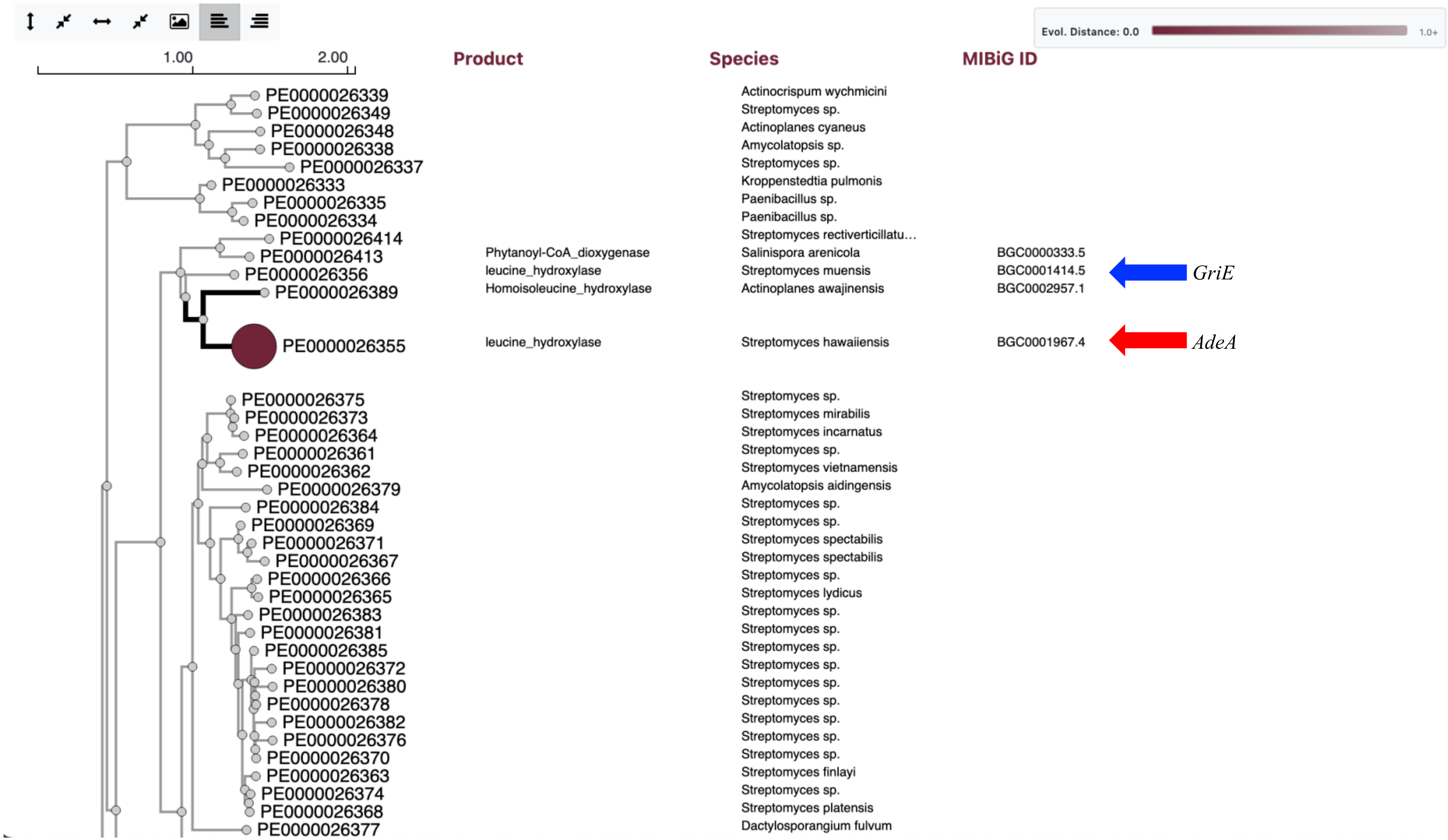

Figure 8: Detail from tree placement ADEP biosynthesis gene *AdeA* in PhyloNaP. *AdeA* and the functionally related *GriE* from Streptomyces muensis are highlighted with arrows.

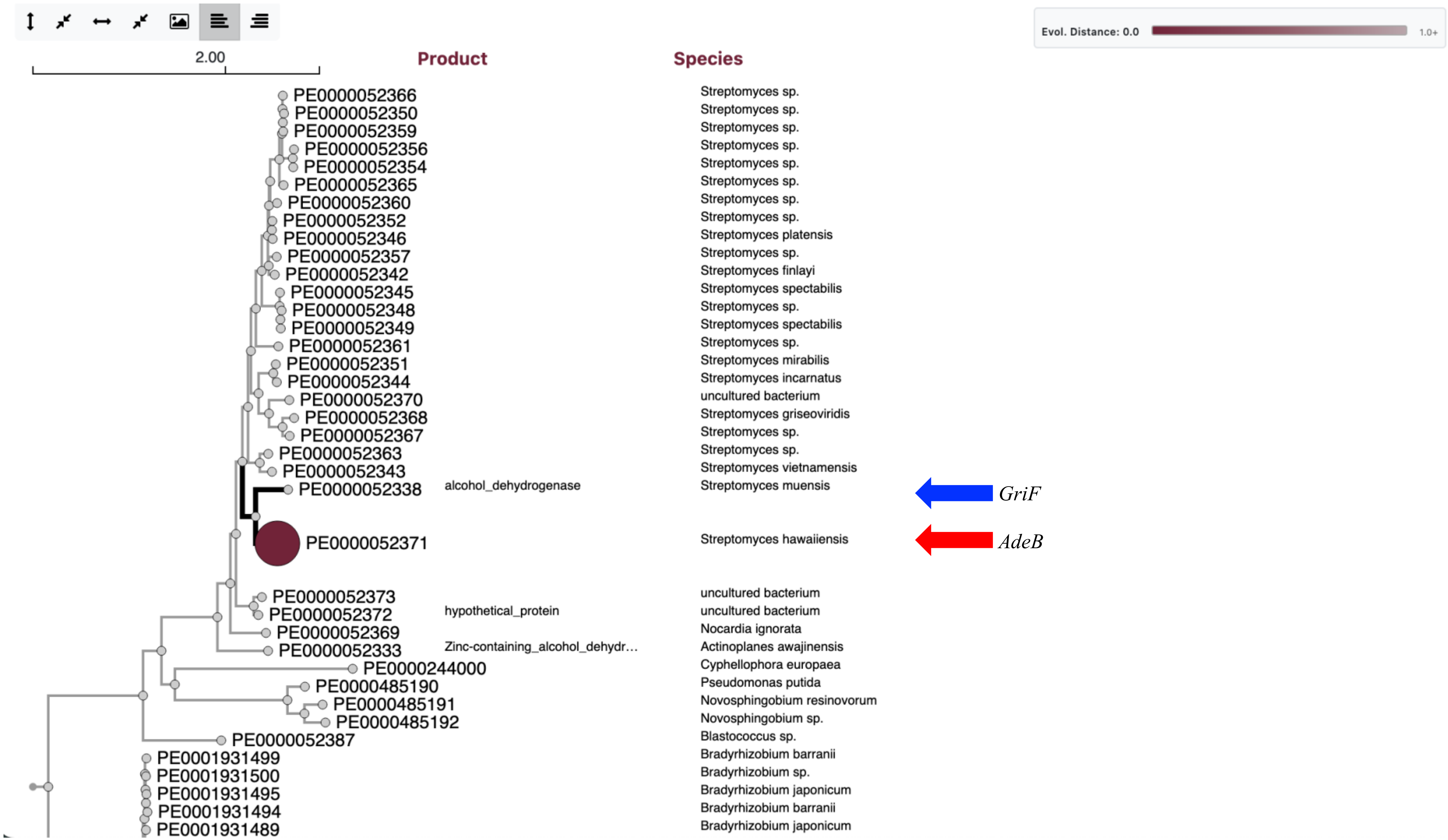

Figure 9: Detail from tree placement ADEP biosynthesis gene *AdeB* in PhyloNaP. *AdeB* and the functionally related *GriF* from Streptomyces muensis are highlighted with arrows.

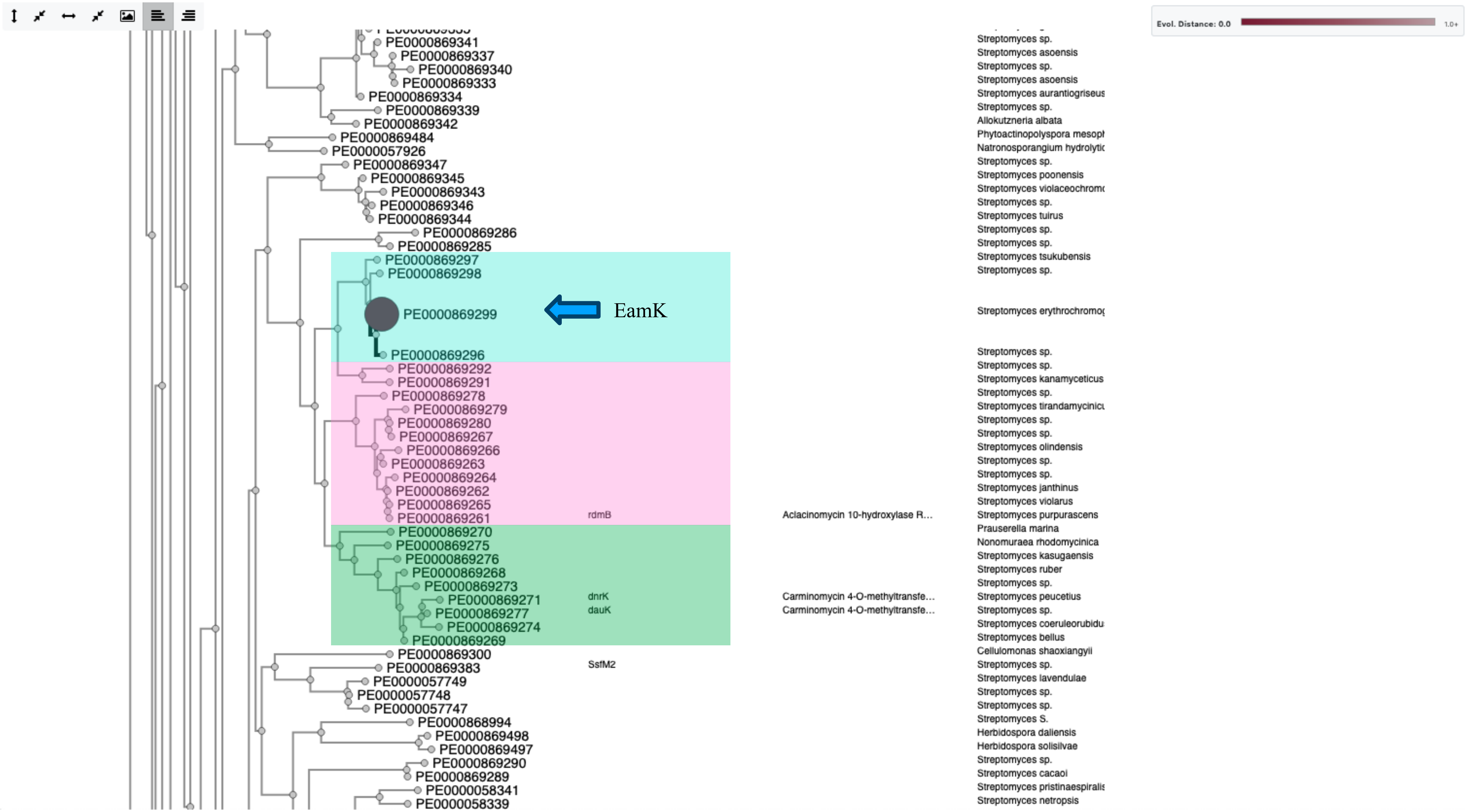

Figure 10: Detail from tree placement of EamK from from the komodoquinone B gene cluster in *Streptomyces erythrochromogenes*. Color codes reflect different functions of mehthytransferase-like genes respective adopted from Grocholski et al. 2019. Green: 4-O methylation & 10-decarboxylation, light pink: 10-hydroxylation, orange: 10-decarboxylation.

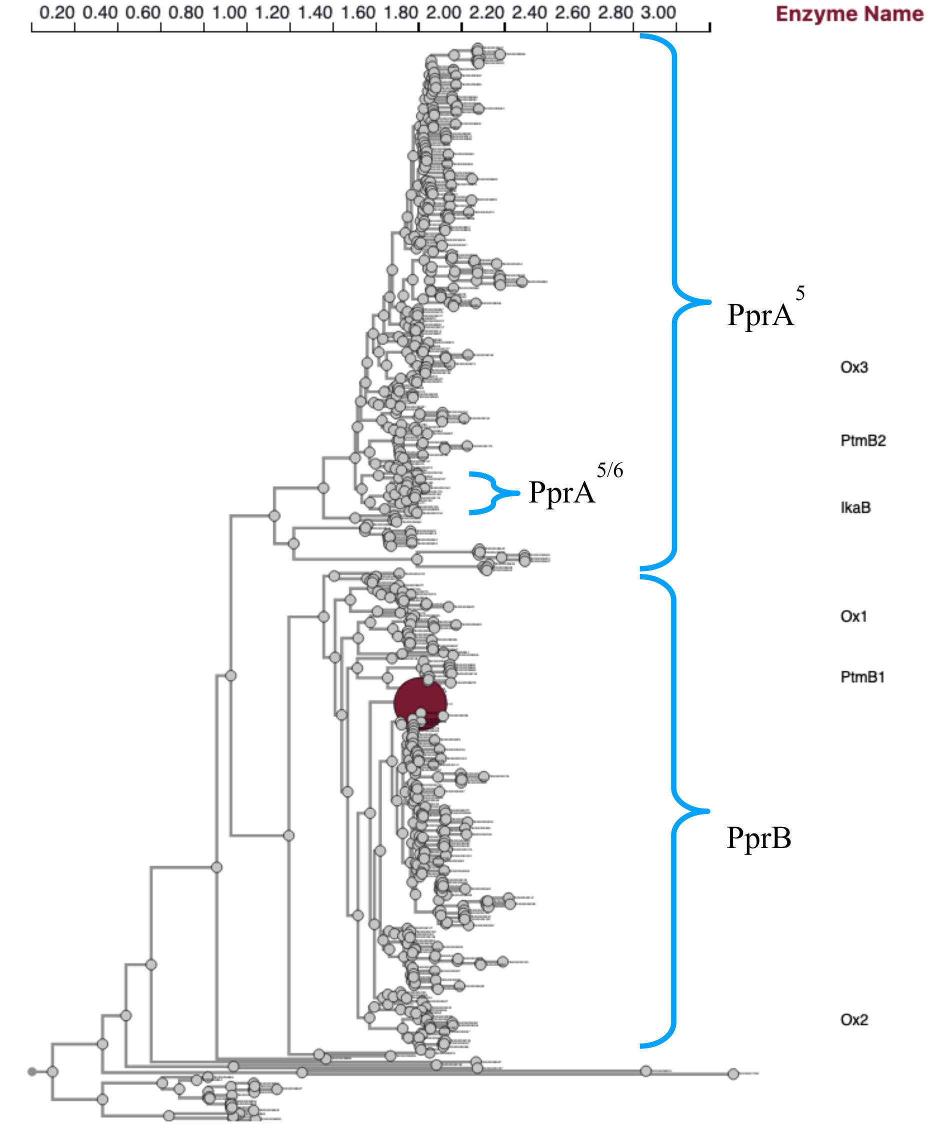

Figure 11: Placement of FtdC from the frontalamide B biosynthetic gene cluster in *Streptomyces sp.* SPB78. Clade description as PprA[5], PprA[5/6] and PprB is shown according to the reference tree from Harper et al. 2024.

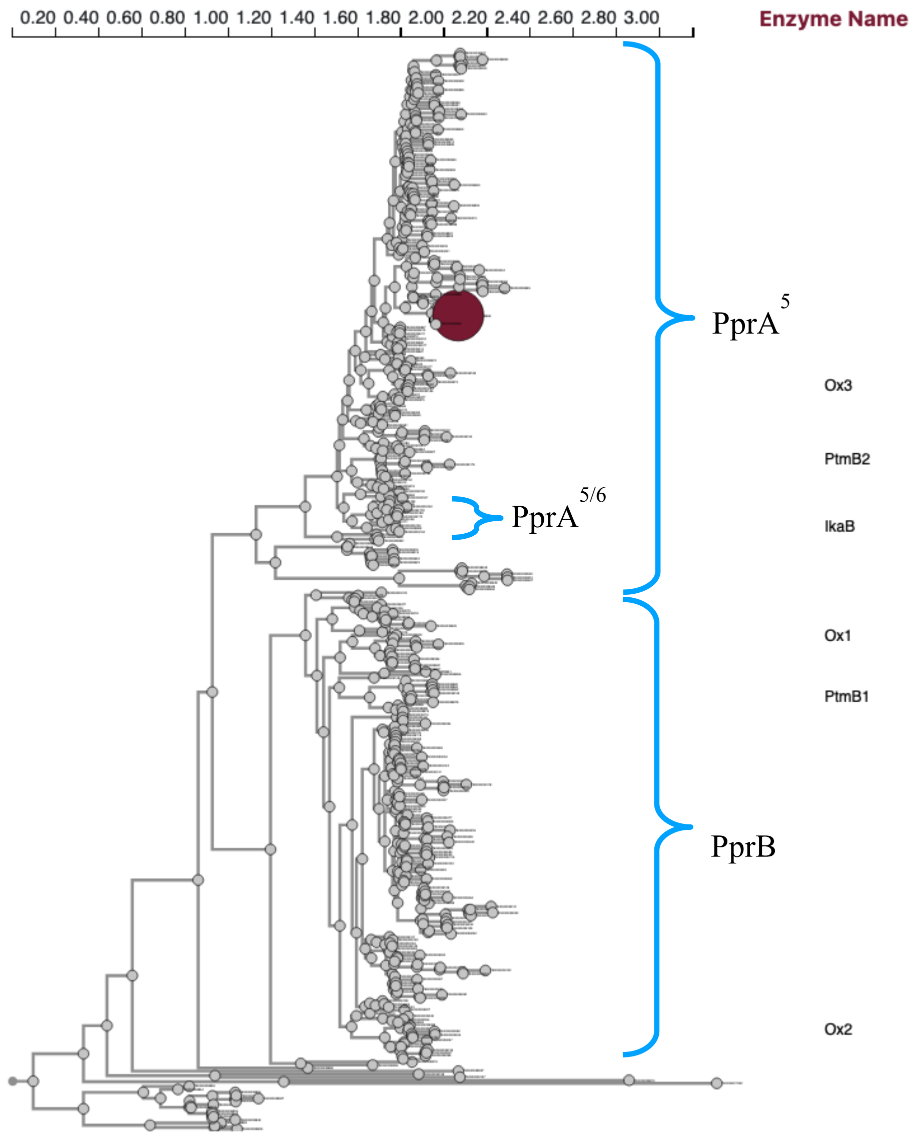

Figure 12: Placement of FtdD from the frontalamide B biosynthetic gene cluster in *Streptomyces sp.* SPB78. Claddescription as PprA[5], PprA[5/6] and PprB is shown according to the referenc tree from Harper et al. 2024.

Database functional overview

The functional diversity of datasets (Figure 1B in the article) was assessed in two complementary ways.

Functional categories from eggNOG.

To provide an overview of functional diversity, one representative sequence was selected from each dataset. The representative was defined as the central sequence, i.e. the sequence with the minimal sum of evolutionary distances to all other sequences in the phylogenetic tree. Representatives were annotated using eggNOG-mapper (Cantalapiedra et al. 2021) using EggNOG 6.0 database (Hernández-Plaza et al. 2023) assign general functional roles.

Datasets were classified into five major categories based on COG functional assignments: biosynthetic, transporter, regulator, other, and unclassified. Proteins associated with amino acid, nucleotide, lipid, coenzyme, energy, or secondary metabolite metabolism were classified as biosynthetic. Transporter proteins included those linked to inorganic ion or carbohydrate transport. Sequences annotated under transcription or signal transduction were assigned to the regulator group. Proteins with functions related to primary metabolism, resistance, secretion, or general cellular processes were classified as other, while sequences without a confident assignment remained unclassified. To improve transporter identification and prevent misclassification, the protein description field was screened for transporter-related terms, including “ABC transporter”, “major facilitator”, “MFS”, “permease”, “transporter”, “channel protein”, “importer”, and “exporter”. Matches were reclassified as transporters when appropriate.

When multiple COG categories were assigned to a single sequence, sequence were classified to one of the functional groups followed a hierarchical rule: transporter > regulator > biosynthesis > other > unclassified. This procedure ensured consistent categorization across datasets.

Superfamily-based overview.

rank	superfamily	dataset_count
1	NAD(P)-binding Rossmann-fold domains	1561
2	alpha/beta-Hydrolases	977
3	FAD/NAD(P)-binding domain	647
4	Nucleotide-binding domain	633
5	S-adenosyl-L-methionine-dependent methyltransferases	578
6	Acetyl-CoA synthetase-like	526
7	P-loop containing nucleoside triphosphate hydrolases	482

8	MurCD N-terminal domain	427
9	Thioesterase/thiol ester dehydrase-isomerase	328
10	ACP-like	305
11	PLP-dependent transferases	304
12	Thiolase-like	298
13	Aldolase	238
14	Glyoxalase/Bleomycin resistance protein/Dihydroxybiphenyl dioxygenase	236
15	Terpenoid synthases	212
16	Clavaminate synthase-like	208
17	Radical SAM enzymes	202
18	Ribulose-phoshate binding barrel	188
19	CoA-dependent acyltransferases	183
20	FMN-linked oxidoreductases	178

Supplementary Table 2. Largest enzyme superfamilies represented in the PhyloNaP database. Dataset count shows the number of datasets within the superfamily.

In parallel, the superfamily annotations previously obtained during the dataset-generation stage (via HMMER searches against the Superfamily v1.75 profiles) were retrieved and used to highlight dominant enzyme families. Datasets sharing identical superfamily assignments were grouped, and superfamilies represented by more than 100 datasets were manually inspected. Their functional roles were then assigned into the same five major categories used above. The largest biosynthetic superfamilies are shown in Figure 1B (right panel). A complete list of the top 21 superfamilies, together with their dataset counts, is provided in Supplementary Table 2.

MiBiG database coverage

The overlap between MiBiG and PhyloNaP was assessed by mapping MiBiG sequences to the PhyloNaP database using available functional category annotations (figure 13). Due to the limited availability of such annotations in MiBiG, a substantial proportion of sequences could not be classified in this analysis.

Despite this limitation, approximately 65% of MiBiG sequences are represented in PhyloNaP. Notably, coverage is substantially higher for enzyme classes that constitute the primary focus of PhyloNaP: tailoring enzymes and precursor biosynthesis enzymes are represented at 82% and 78%, respectively. These results demonstrate strong coverage of functionally relevant biosynthetic enzymes within the database.

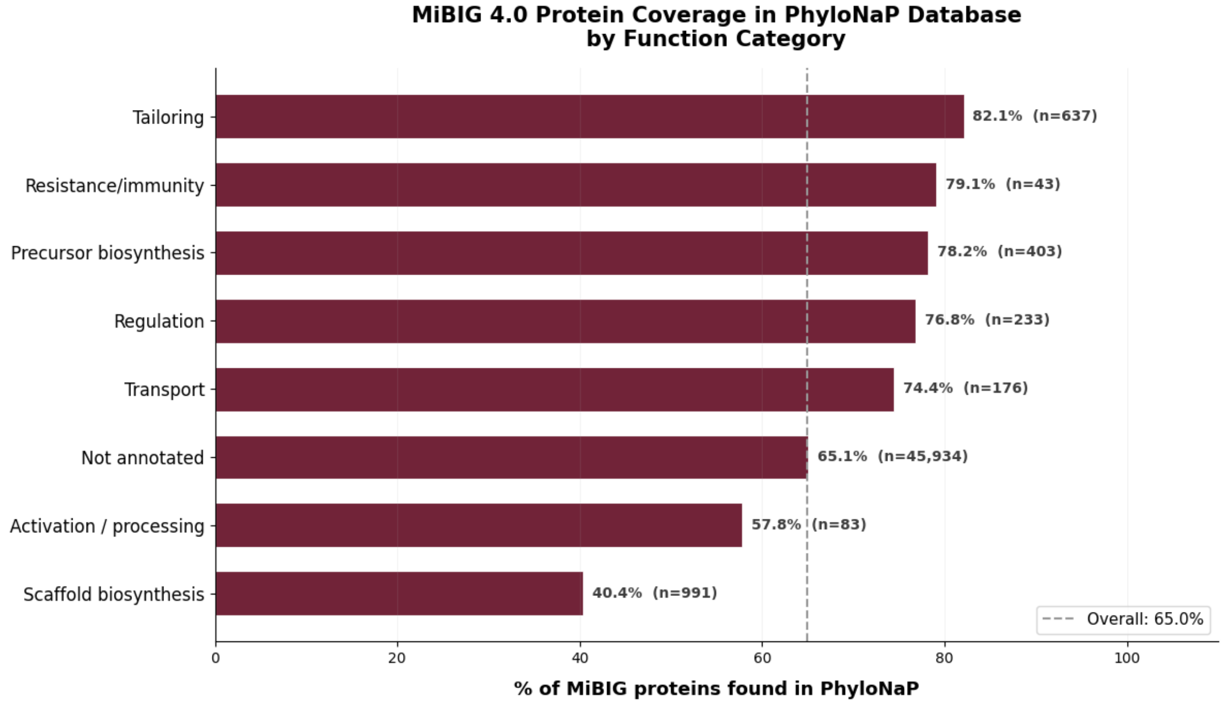

Figure 13. The overview of the MIBiG functional categories coverage.

A workflow example

As an additional illustration, we tested the placement of a flavin-dependent halogenase (A0A1L1QK36 in UniProt). The analysis page shows placements onto two reference datasets (Supplementary figure 1). By default, only the best-scoring dataset is displayed, but the user can toggle a button to view alternatives. Within a tree, the query sequence may be placed onto a leaf or an internal node, and sometimes multiple placement options are reported. Typically, the more divergent a query sequence is from the reference tree sequences, the more alternative placements are reported, reflecting increased uncertainty (Matsen et al. 2012; Barbera et al. 2019).

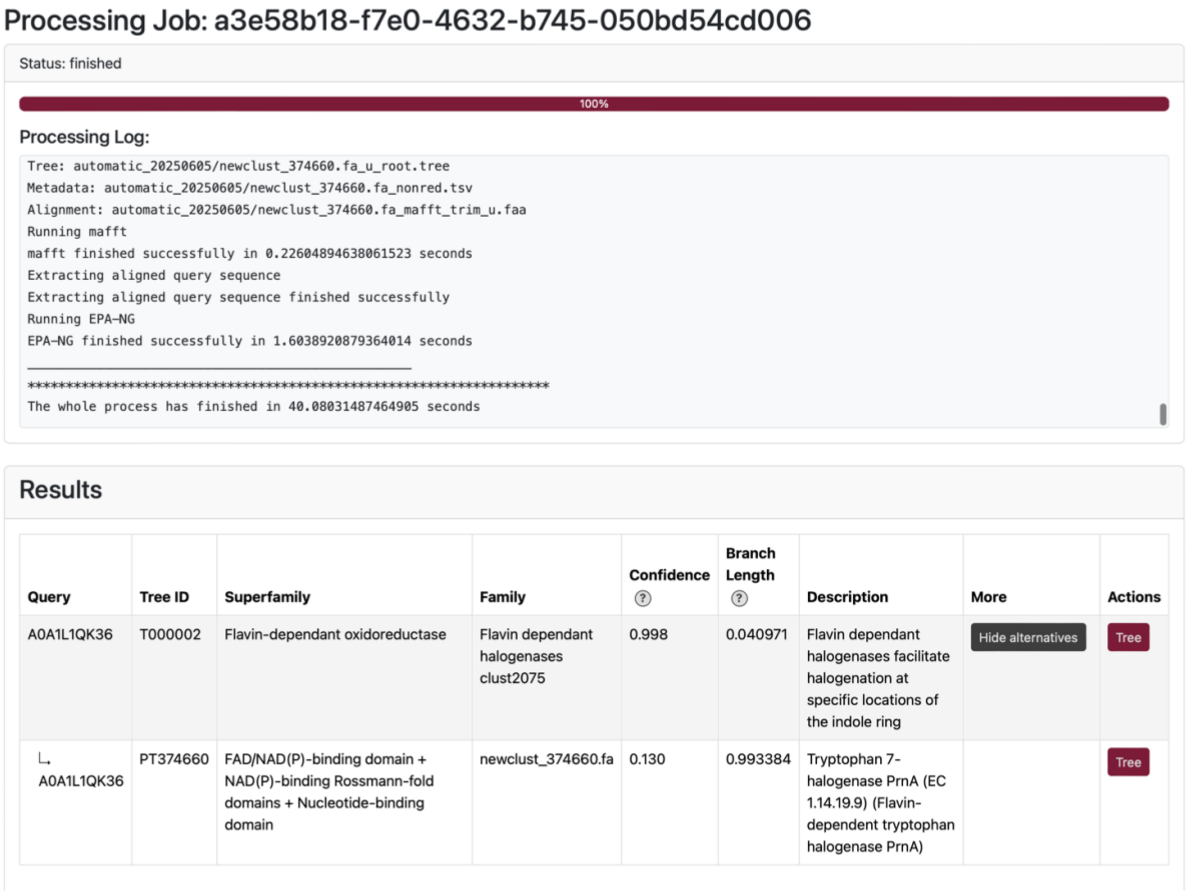

Figure 14. Query sequence placements on two reference datasets.

The confidence column reports the likelihood weight ratio (LWR) of the best placement for each reference dataset. LWR measures how strongly the data support a given placement relative to all other possible placements of the same query sequence within that dataset. All LWRs for the query within one tree sum to 1. In our example, the placement in the prioritized reference dataset has an LWR of 0.998, while the best placement in the second reference dataset has an LWR of 0.130, indicating that the first dataset provides much stronger support.

The branch length indicates the evolutionary distance between the query and the node of placement based on the alignment and substitution model. A short branch length suggests closer similarity, which makes it more reasonable to infer function from the surrounding clade. Conversely, a long branch

length suggests that the alignment places the query far from its nearest reference, and any inference becomes more tentative. In this case, the placement on the prioretised reference dataset has a short branch length (≈0.04 substitutions per site), supporting a reliable placement, while in the second reference dataset the branch length is nearly 1, signaling much weaker support.

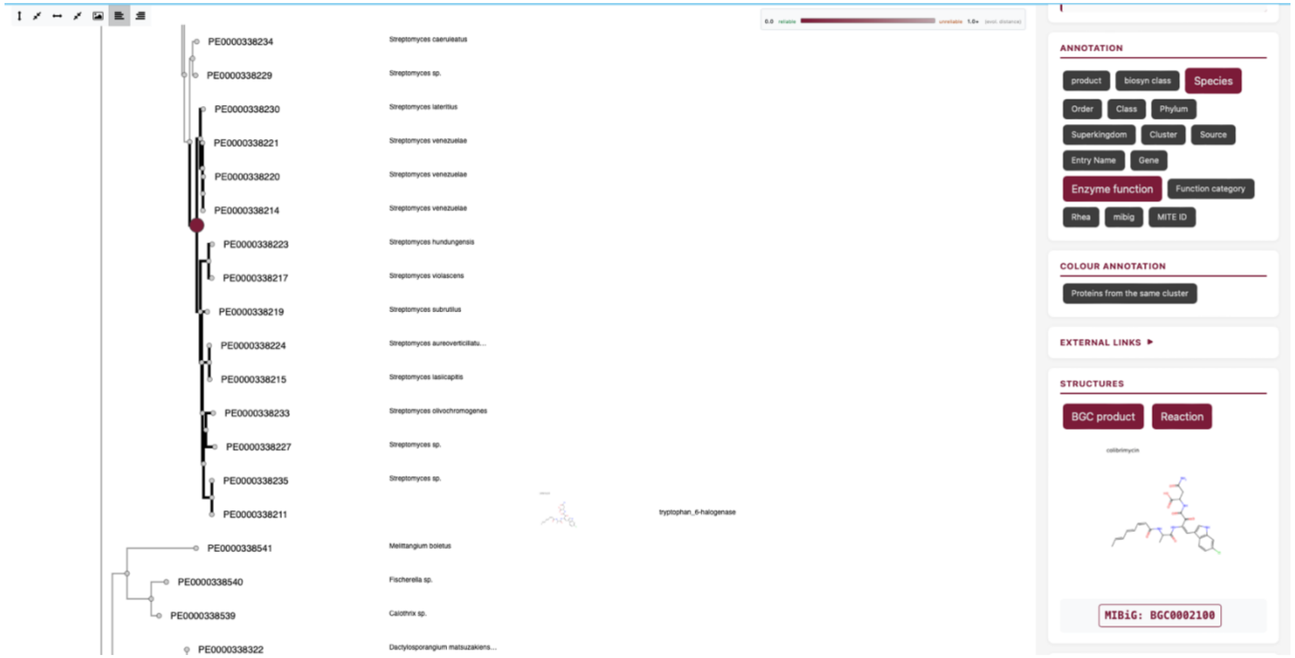

Figure 15. Placement node (red circle) and corresponding clade (highlighted in red) with Annotations and Metadata summary.

Let us now inspect the dataset with the reliable placement. The placement node is marked in red circle, and the clade defined by this node is highlighted in the same color (Figure 15). All available annotations for this clade are summarized in the “Metadata summary” field, which shows how consistent each type of annotation is. From this, we see that nearly all proteins in the clade, except one, are associated with nonribosomal peptide synthetase (NRPS) biosynthetic gene clusters, indicating that their substrates are amino acids or peptides. All proteins in the clade come from species of the genus *Streptomyces*. One enzyme carries a functional annotation as a tryptophan-6 halogenase.

To investigate further, we can click the “Enzyme function” button in the metadata panel to display this annotation directly. Additionally, the “Structures” field provides the option to visualize BGC products: by selecting it, images of natural products from the MIBiG database, in whose biosynthesis the proteins are involved, appear. In this case, one protein in the clade has both a functional annotation and a linked product structure, allowing us to cross-validate the assignment and confirm that it is indeed a tryptophan-6 halogenase.

A.

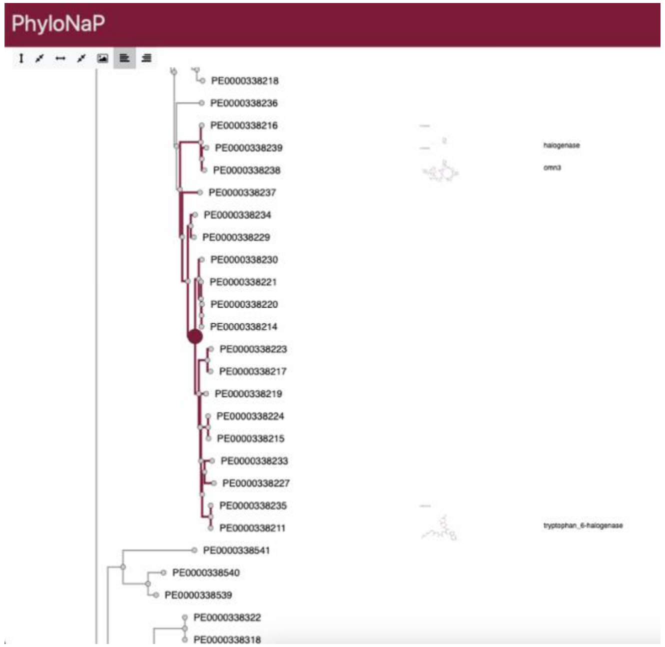

PhyloNaP
PE0000338218
PE0000338236
PE0000338216
PE0000338239
halogenase
PE0000338238
omn3
PE0000338237
PE0000338234
PE0000338229
PE0000338230
PE0000338221
PE0000338220
PE0000338214
PE0000338223
PE0000338217
PE0000338219
PE0000338224
PE0000338215
PE0000338233
PE0000338227
PE0000338235
PE0000338211
tryptophan_6-halogenase
PE0000338541
PE0000338540
PE0000338539
PE0000338322
PE0000338318

B.

.
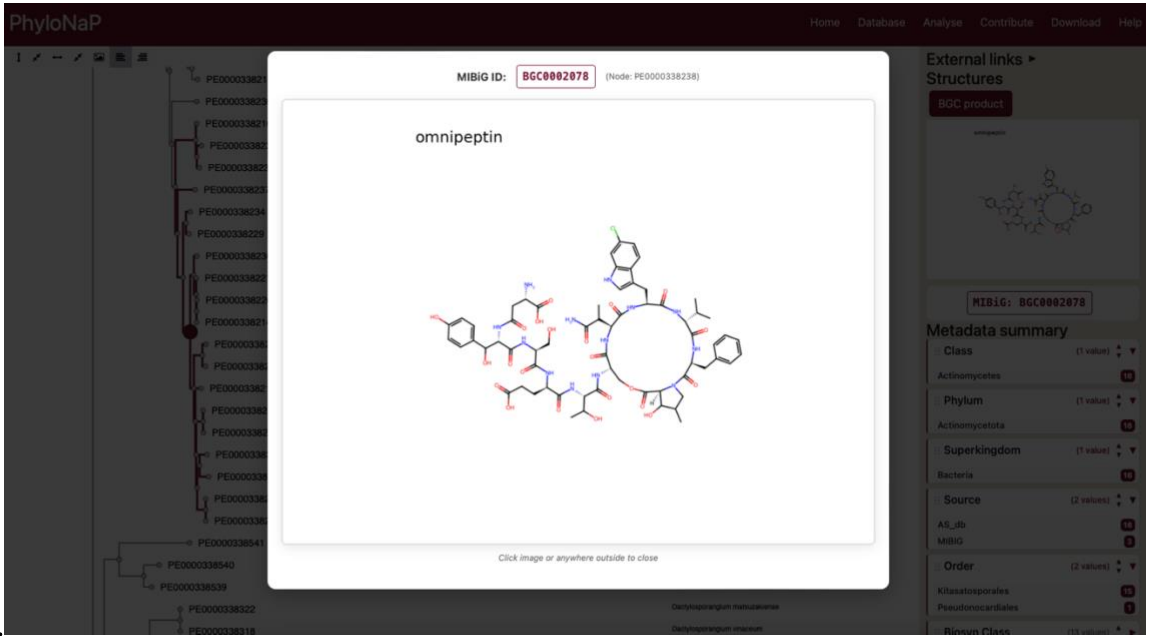

Supplementary Figure 16. A. Smallest ancestral clade including additional annotated proteins (highlighted in red). B. Enlarged chemical structure of a natural product associated with an additional annotated protein, showing a halogen substituent at position 6 of tryptophan.

Since no other detailed annotations are available within the clade itself, we can examine the nearest annotated ancestral node (Supplementary Figure 16A), the clade is indicated in red. Inspection of the proteins branching from this node reveals another natural product that clearly carries a halogen substituent at position 6 of tryptophan (Supplementary Figure 16B). Even though the text annotation of the associated enzyme function does not specify the substitution site, the chemical structure image confirms it.

In summary, the reliable placement, the short branch length, and the agreement between phylogenetic and chemical evidence all point to the same conclusion: the query protein most likely carries out the same reaction. The clade, defined by their common ancestor, shows consistent functional annotations, supporting that our enzyme catalyzes halogenation of tryptophan at position 6. This combination of visual and contextual information enables researchers to infer substrate class and biochemical role, guiding downstream functional studies.

References

Bansal, Parit, Anne Morgat, Kristian B Axelsen, et al. 2022. “Rhea, the Reaction Knowledgebase in 2022.” *Nucleic Acids Research* 50 (D1): D693–700. https://doi.org/10.1093/nar/gkab1016.

Barbera, Pierre, Alexey M Kozlov, Lucas Czech, et al. 2019. “EPA-Ng: Massively Parallel Evolutionary Placement of Genetic Sequences.” *Systematic Biology* 68 (2): 365–69. https://doi.org/10.1093/sysbio/syy054.

Blin, Kai, Simon Shaw, Marnix H. Medema, and Tilmann Weber. 2024. “The antiSMASH Database Version 4: Additional Genomes and BGCs, New Sequence-Based Searches and More.” *Nucleic Acids Research* 52 (D1): D586–89. https://doi.org/10.1093/nar/gkad984.

Boutet, Emmanuel, Damien Lieberherr, Michael Tognolli, Michel Schneider, and Amos Bairoch. 2007. “UniProtKB/Swiss-Prot.” *Methods in Molecular Biology (Clifton, N.J.)* 406: 89–112. https://doi.org/10.1007/978-1-59745-535-0_4.

Bryant, David, and Michael Charleston. 2018. “MAD Roots for Large Trees.” arXiv:1811.03174. Preprint, arXiv, November 7. https://doi.org/10.48550/arXiv.1811.03174.

Cantalapiedra, Carlos P, Ana Hernández-Plaza, Ivica Letunic, Peer Bork, and Jaime Huerta-Cepas. 2021. “eggNOG-Mapper v2: Functional Annotation, Orthology Assignments, and Domain Prediction at the Metagenomic Scale.” *Molecular Biology and Evolution* 38 (12): 5825–29. https://doi.org/10.1093/molbev/msab293.

Capella-Gutiérrez, Salvador, José M. Silla-Martínez, and Toni Gabaldón. 2009. “trimAl: A Tool for Automated Alignment Trimming in Large-Scale Phylogenetic Analyses.” *Bioinformatics* 25 (15): 1972–73. https://doi.org/10.1093/bioinformatics/btp348.

Cui, Zhanzhao, et al. (2025), ‘Characterization of the Postaglycone Modifications in Ristomycin Biosynthesis’, *ACS Chemical Biology*, 20/7: 1764–74, https://doi.org/10.1021/acschembio.5c00280.

Grocholski, Thadée, et al. (2019), ‘Evolutionary Trajectories for the Functional Diversification of Anthracycline Methyltransferases’, *ACS Chemical Biology*, 14/5: 850–6, https://doi.org/10.1021/acschembio.9b00238.

Hansen, Mathias H., et al. (2023), ‘Resurrecting Ancestral Antibiotics: Unveiling the Origins of Modern Lipid II Targeting Glycopeptides’, *Nature Communications*, 14/1, https://doi.org/10.1038/s41467-023-43451-4.

Harper, Christopher P., et al. (2024), ‘Critical Analysis of Polycyclic Tetramate Macrolactam Biosynthetic Gene Cluster Phylogeny and Functional Diversity’, *Applied and Environmental Microbiology*, 90/6, https://doi.org/10.1128/AEM.00600-24/SUPPL_FILE/AEM.00600-24-S0001.PDF.

Hastings, Janna, Gareth Owen, Adriano Dekker, et al. 2016. “ChEBI in 2016: Improved Services and an Expanding Collection of Metabolites.” *Nucleic Acids Research* 44 (D1): D1214-9. https://doi.org/10.1093/nar/gkv1031.

Hernández-Plaza, Ana, Damian Szklarczyk, Jorge Botas, et al. 2023. “eggNOG 6.0: Enabling Comparative Genomics across 12 535 Organisms.” *Nucleic Acids Research* 51 (D1): D389–94. https://doi.org/10.1093/nar/gkac1022.

Katoh, Kazutaka, and Daron M. Standley. 2013. “MAFFT Multiple Sequence Alignment Software Version 7: Improvements in Performance and Usability.” *Molecular Biology and Evolution* 30 (4): 772–80. https://doi.org/10.1093/molbev/mst010.

Landrum, Greg, Paolo Tosco, Brian Kelley, et al. 2020. *Rdkit/Rdkit: 2020_03_1 (Q1 2020) Release*. V. Release_2020_03_1. Zenodo, released March 29. https://doi.org/10.5281/zenodo.3732262.

Matsen, Frederick A., Noah G. Hoffman, Aaron Gallagher, and Alexandros Stamatakis. 2012. “A Format for Phylogenetic Placements.” *PLoS ONE* 7 (2): e31009. https://doi.org/10.1371/journal.pone.0031009.

O’Leary, Nuala A., Eric Cox, J. Bradley Holmes, et al. 2024. “Exploring and Retrieving Sequence and Metadata for Species across the Tree of Life with NCBI Datasets.” *Scientific Data* 11 (1): 732. https://doi.org/10.1038/s41597-024-03571-y.

Paccagnella, Davide, Caner Bagci, Athina Gavriilidou, and Nadine Ziemert. 2025. “PanBGC: A Pangenome-Inspired Framework for Comparative Analysis of Biosynthetic Gene Clusters.” Preprint, bioRxiv, August 11. https://doi.org/10.1101/2025.08.11.669102.

Pandurangan, Arun Prasad, Jonathan Stahlhacke, Matt E Oates, Ben Smithers, and Julian Gough. 2019. “The SUPERFAMILY 2.0 Database: A Significant Proteome Update and a New Webserver.” *Nucleic Acids Research* 47 (D1): D490–94. https://doi.org/10.1093/nar/gky1130.

Price, Morgan N., Paramvir S. Dehal, and Adam P. Arkin. 2010. “FastTree 2--Approximately Maximum-Likelihood Trees for Large Alignments.” *PloS One* 5 (3): e9490. https://doi.org/10.1371/journal.pone.0009490.

Steinegger, Martin, and Johannes Söding. 2017. “MMseqs2 Enables Sensitive Protein Sequence Searching for the Analysis of Massive Data Sets.” *Nature Biotechnology* 35 (11): 1026–28. https://doi.org/10.1038/nbt.3988.

The UniProt Consortium. 2025. “UniProt: The Universal Protein Knowledgebase in 2025.” *Nucleic Acids Research* 53 (D1): D609–17. https://doi.org/10.1093/nar/gkae1010.

Thomy, Dhana, et al. (2019), ‘The ADEP Biosynthetic Gene Cluster in Streptomyces Hawaiiensis NRRL 15010 Reveals an Accessory ClpP Gene as a Novel Antibiotic Resistance Factor’, *Applied and Environmental Microbiology*, 85/20: 1–18, https://doi.org/https://doi.org/10.1128/AEM.01292-19 334.207.

Zdouc, Mitja M., David Meijer, Friederike Biermann, et al. 2024. “The Minimum Information about a Tailoring Enzyme/Maturase Data Standard for Capturing Natural Product Biosynthesis.” Preprint, ChemRxiv, April 9. https://doi.org/10.26434/chemrxiv-2024-78mtl.

Zdouc, Mitja M, Kai Blin, Nico L L Louwen, et al. 2024. “MIBiG 4.0: Advancing Biosynthetic Gene Cluster Curation through Global Collaboration.” *Nucleic Acids Research*, December 9, gkae1115. https://doi.org/10.1093/nar/gkae1115.
